# Integrative Taxonomy of *Nuchequula longicornis* (Teleostei: Leiognathidae) from Chinese Waters: Morphological Analysis, Mitogenomic Characterization, and Phylogenetic Implications

**DOI:** 10.3390/biology15030260

**Published:** 2026-01-30

**Authors:** Ning-Ya Yang, Sheng Zeng, De-Yuan Yang, Jun-Sheng Zhong, Pan Liu, Xiao-Dong Wang, Jia-Jie Chen

**Affiliations:** 1 Shanghai Universities Key Laboratory of Marine Animal Taxonomy and Evolution, Shanghai Ocean University, Shanghai 201306, China; 15234038315@163.com (N.-Y.Y.); jszhong@shou.edu.cn (J.-S.Z.); 2 State Key Laboratory of Marine Environmental Science, College of Ocean and Earth Sciences, Xiamen University, Xiamen 361102, China; 33120231152326@stu.xmu.edu.cn (S.Z.); deyuanyang92@163.com (D.-Y.Y.); 3 Conversation and Research Center for Collections, Shanghai Natural History Museum, Branch of Shanghai Science and Technology Museum, Shanghai 200041, China; liup@sstm.org.cn; 4 East China Sea Fisheries Research Institute, Fisheries Science of Chinese Academy, Shanghai 200090, China

**Keywords:** mitochondrial genome, *Nuchequula longicornis*, Leiognathidae, phylogenetic tree, South China Sea

## Abstract

The ponyfish genus *Nuchequula* is presents taxonomic challenges within Leiognathidae due to morphological complexities and a lack of genomic resources. However, the absence of a complete mitochondrial genome for *Nuchequula longicornis* has hindered comprehensive phylogenetic comparisons. Here, we employed an integrative approach combining detailed morphology with molecular validation via 16S rRNA gene sequencing to confirm the identity of specimens from the Beibu Gulf. Furthermore, we present the first complete mitogenome of *N. longicornis*. This circular 16,514 bp molecule exhibits a typical gene order, a moderate AT bias (54.8%), and strong purifying selection across all protein-coding genes. Phylogenomic analysis based on the complete mitogenome reliably resolved the position of *N. longicornis* within Leiognathidae, contributing to the discussion on the phylogenetic relationships and potential monophyly of *Nuchequula* and contributing to the understanding of intra-familial relationships. Our study provides an essential mitogenomic resource for *N. longicornis* and offers insights into mitochondrial evolutionary patterns within this family.

## Simple Summary

To accurately identify species and trace their evolutionary history, scientists combine detailed physical examination with genetic analysis. For the poorly studied ponyfish *Nuchequula longicornis*, we first conducted a thorough morphological study of specimens from the Beibu Gulf. We then employed the 16S rRNA gene as a molecular tool to genetically confirm their identity by comparing them to known references. Finally, we decoded and analyzed the fish’s complete mitochondrial genome (“mitogenome”) for the very first time. This new genetic blueprint details its structure and clarifies its evolutionary relationships within the ponyfish family. Beyond providing a vital resource for future identification and conservation, our integrative approach—morphology plus molecules—offers fresh insights for refining the classification of this group.

## 1. Introduction

The ponyfish *Nuchequula longicornis* is characterized by its distinct elongated second dorsal-fin spine and nuchal blotch [1]. First described from the Gulf of Thailand [2], it has since been recorded in Malaysia, Vietnam, and Myanmar [3,4,5]. This species belongs to *Nuchequula*, a genus comprising approximately seven recognized species (four of which occur in Chinese waters: *Nuchequula blochii*, *N. gerreoides*, *N. mannusella*, and *N. nuchalis* [1,6,7]). The genus has a complex taxonomic history involving multiple synonyms, and despite significant revisions, species delimitation within it remains challenging [1,2,7]. Although sequences for partial mitochondrial genes (e.g., 16S and 12S rRNA) are available for *N. longicornis*, a key piece of molecular data—the complete mitochondrial genome—remains unavailable for this species, limiting the resolution genomic comparisons and utility of mitogenomic-scale phylogenetic analyses.

The lack of complete mitogenomic data for *N. longicornis* is particularly notable within the broader context of phylogenetic research on the family Leiognathidae. The phylogenetic framework of this family has been progressively refined: initial studies used single mitochondrial genes like 16S rRNA to resolve broad relationships [8]; subsequent regional phylogenetic surveys applied these markers in areas such as the South China Sea [9] and Peninsular Malaysia [10]; multi-locus analyses combining mitochondrial and nuclear genes later established a more robust phylogeny and explored drivers of diversification like sexual selection [11]; and recently, DNA barcoding (e.g., COI, 16S) has become a standard tool for species identification and assessment of genetic variation across regions including Indian [12,13] and Chinese waters [14].

In contrast, phylogenetic understanding within the genus *Nuchequula* has lagged behind and remains heavily reliant on morphology. The genus has been a persistent focus of taxonomic revision due to complexities in species delimitation driven by morphological conservatism and plasticity [1,2]. For instance, the validity and boundaries of species such as *N. gerreoides* and *N. mannusella* remain debated [6,7,15]. While comprehensive taxonomic revisions and regional reviews based on morphology have clarified some species boundaries and distributions, a robust molecular phylogeny for the genus is still lacking. This deficiency is exemplified at the genomic level: although a complete mitochondrial genome is available for a congener (*N. nuchalis*), it remains absent for *N. longicornis* and other congeners. The absence of such data hinders precise phylogenetic placement of these species and constrains detailed comparative mitogenomic studies within *Nuchequula*.

To resolve such taxonomic and phylogenetic questions, integrative approaches that combine detailed morphology with molecular data are fundamental in ichthyology. Therefore, to confirm the identity of our specimens as *Nuchequula longicornis* from the Beibu Gulf (given observed variations in spine length and the absence of local reference material) and to simultaneously address the critical lack of genomic data, we employed an integrative approach. For the primary molecular identification, we selected the mitochondrial 16S ribosomal RNA (16S rRNA) gene. This choice was primarily based on its established role as a conventional and effective marker for phylogenetic inference and species delineation within the Leiognathidae family [9,13], ensuring our results are directly comparable to the existing body of literature. Additionally, robust COI barcode references for *N. longicornis* are currently lacking, further supporting the use of 16S rRNA as the most practical molecular tool for initial validation. The relatively conserved sequence and ease of amplification with universal primers allowed for efficient validation against existing references (e.g., from Vietnam), serving as a crucial first step prior to complete mitogenome characterization. This strategic use of 16S rRNA (leveraging its particular utility in groups with established reference frameworks and for reliable amplification) is also exemplified by its successful application to identify commercial sparid species [16] and processed eel species [17].

Therefore, to decisively address the critical absence of mitogenomic data for *N. longicornis*, this study was designed with the following objectives: (1) to provide a detailed morphological description confirming the first record of *Nuchequula longicornis* in Chinese waters; (2) to perform molecular validation of species identity using the 16S rRNA gene; (3) to sequence, annotate, and characterize the complete mitochondrial genome of *N. longicornis* for the first time; and (4) to determine its phylogenetic position within Leiognathidae through phylogeny analysis based on this complete mitogenomic dataset. By establishing this foundational genetic resource and clarifying evolutionary relationships patterns, this work aims to facilitate future taxonomic and comparative studies within the genus *Nuchequula*.

## 2. Materials and Methods

### 2.1. Sample Collection and DNA Extraction

**Figure 1:**
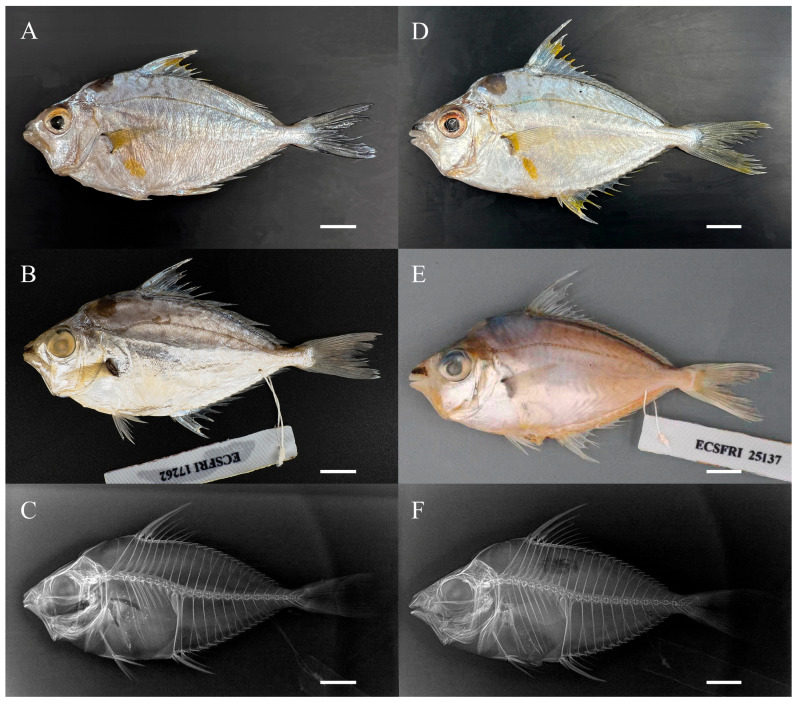
Left lateral views of *Nuchequula longicornis*. (**A**–**C**). Voucher no. ECSFRI 17262; GenBank accession no. PX227129; 79.53 mm SL. (**A**) Fresh coloration after thawing, mirror-reversed from right lateral view. (**B**) Preserved specimen (fixed in 95% ethanol, stored in 70% ethanol). (**C**) Radiograph. (**D**–**F**) Voucher no. ECSFRI 25137; GenBank accession no. PX227129; 79.53 mm SL. (**D**) Fresh coloration. (**E**) Preserved specimen (fixed in 10% formalin, transferred to 70% ethanol). (**F**) Radiograph. Scale bars = 10 mm. Photos by Jia-Jie Chen.

All specimens are deposited at the East China Sea Fisheries Research Institute (ECSFRI), Shanghai, China. The material examined includes nine specimens of *Nuchequula longicornis* (voucher nos.: ECSFRI 17255–17263; Figure 1A–C) were collected from the Beibu Gulf, near Hainan Island, in April 2022. Six specimens of *N. longicornis* (voucher nos.: ECSFRI 25135–25140; Figure 1D–F) were collected from Qiaogang Port, Beibu Gulf, in January 2024.

Prior to DNA isolation, the specimen surface was cleaned with 100% ethanol. Approximately 10 × 10 mm^2^ of muscle tissue was excised from below the right dorsal fin for genomic DNA extraction. Total genomic DNA was extracted using the TIANamp Genomic DNA Kit (TIANGEN, Beijing, China). All specimens were fixed in 10% formalin and subsequently transferred to 70% ethanol for long-term preservation. Of these, specimens ECSFRI 17255 and ECSFRI 25137 were selected for mitogenome resequencing.

### 2.2. Mitogenome Sequencing and Assembly

The mitochondrial genomes were sequenced and assembled following established protocols [18,19,20]. Total genomic was used for library construction. DNA libraries were prepared with the Illumina TruSeqTM DNA Sample Preparation Kit (Illumina, San Diego, CA, USA) following manufacturer guidelines, with an average insert size of ~350 bp. The quality and concentration of the DNA libraries were assessed using an Agilent 2100 Bioanalyzer (Agilent Technologies, Santa Clara, CA, USA) and Qubit Fluorometer (Thermo Fisher Scientific, Waltham, MA, USA), respectively. Shanghai Majorbio Bio-pharm Technology Co., Ltd. (Shanghai, China) conducted sequencing on a DNBSEQ-T7 platform, generating 150 bp paired-end reads yielding ~5 Gb raw data per sample. The Illumina TruSeq kit is compatible with the DNBSEQ-T7 platform for generating high-quality sequencing libraries, as both systems utilize a similar bridge amplification-based clustering technology. Data preprocessing involved Fastp v0.23.2 [21] with default parameters (including removal of adapter sequences and low-quality reads) and quality assessment using FastQC v0.12.1 (http://www.bioinformatics.babraham.ac.uk/projects/fastqc/, accessed on 10 January 2025). Mitogenome assembly employed the FastMitoAssembler pipeline, augmented by GetOrganelle v1.7.6.1 [22] and NovoPlasty v4.3.1 [23].

### 2.3. Mitogenome Annotation and Sequence Analyses

The complete mitochondrial genome was annotated using MITOS2 2.1.10+galaxy0 [24] and MitoZ v3.6 [25], with manual validation in Geneious v2021.0.3. PhyloSuite v1.2.3 [26] was employed to quantify base composition, codon usage, and relative synonymous codon usage (RSCU) of protein-coding genes (PCGs). Nucleotide skewness (A + T skew = [A% − T%]/[A% + T%]; G + C skew = [G% − C%]/[G% + C%]) was calculated following Perna and Kocher [27]. Selective pressure analysis through Ka/Ks ratios was conducted for Leiognathidae species mitogenomes using DnaSP 6.0 [28], and results were visualized using Origin 8. Transfer RNA secondary structures were predicted using MITOS2 2.1.10+galaxy0 [24,29] and visualized using Python 3.11-based bioinformatics packages.

Due to identical mitochondrial genomes between voucher specimens ECSFRI 17255 and ECSFRI 25137, the latter (ECSFRI 25137) was selected as the representative for detailed description of the complete mitochondrial genome.

### 2.4. Phylogenetic Analysis Methodology

Initial phylogenetic analysis was conducted based on the 16S rRNA gene using sequences from the complete mitogenome generated in this study together with 99 leiognathid sequences retrieved from NCBI (Table S1). The resulting 16S tree was inferred under the best-fit substitution models GTR+F+I+G4 for Bayesian inference (BI) and TIM2+F+I+I+R3 for maximum likelihood (ML).

Subsequently, a phylogeny of Leiognathidae was reconstructed using a dataset of 46 complete mitogenomes, comprising 37 species, with *Lagocephalus gloveri* and *Amblygobius phalaena* designated as outgroups. Mitogenomic sequences obtained from GenBank (Table S2) were processed using PhyloSuite v1.2.3 [26]. The 13 protein-coding genes (PCGs) were aligned codon-by-codon using MAFFT v7.313 [30], and ambiguously aligned regions were removed using Gblocks v0.91 [31]. To mitigate the potential effects of compositional bias and saturation in mitochondrial sequences, phylogenetic inference was performed on the nucleotide alignment of the 13 PCGs. The best-fit substitution models for the unpartitioned nucleotide alignment using ModelFinder v2.2.0 [32] under the Bayesian Information Criterion (BIC) for ML analysis and the corrected Akaike Information Criterion (AICc) for BI analysis. The selected schemes and models are detailed in Table S3.

A phylogeny of Leiognathidae was reconstructed using a curated dataset derived from 46 publicly available mitogenomes, comprising 37 species (Table S2). To root the tree appropriately within a larger phylogenetic context, *Lagocephalus gloveri* (Tetraodontidae) and *Amblygobius phalaena* (Gobiidae) were selected as primary outgroups, based on established relationships in acanthomorph fishes. Preliminary analyses with more distant outgroups (e.g., Acanthuridae) confirmed that the internal topology and support within Leiognathidae remained stable.

Maximum likelihood analysis was performed with IQ-TREE v2.2.2 under edge-linked partition models and supported with 100,000 ultrafast bootstrap replicates. Bayesian inference was carried out using MrBayes v3.2.7a [33], running two independent analyses for 2 × 10^6^ generations each. Resulting trees were visualized and annotated in iTOL v6 [34].

Given that maximum likelihood bootstrap proportions (ML-BP) and Bayesian posterior probabilities (BI-PP) are distinct statistical measures with different sensitivities to data structure and model assumptions, discrepancies between their values on a given node are not uncommon. ML bootstrap assesses the robustness of a clade to data resampling, whereas BI posterior probability represents the probability of a clade given the model and data. Notable differences, particularly strong support from only one method, can arise from factors such as limited phylogenetic signal at specific nodes, model misspecification, or challenges in MCMC convergence for certain tree regions. Therefore, in the interpretation of our results, clades receiving strong support from both methods (e.g., ML-BP ≥ 70% and BI-PP ≥ 0.95) are considered robust, while those supported by only one method are treated with caution and noted as such in the discussion.

### 2.5. Morphological Study

Morphometric methods followed Kimura et al. [2] and Chakrabarty and Sparks [1]. All counts and measurements were taken from specimens fixed 10% formalin and subsequently preserved in 70% ethanol. The full set of 15 specimens was utilized for standard morphometric measurements and meristic counts. To examine skeletal and dental structures in detail, a subset of two specimens (voucher nos. ECSFRI 25137, 25140) was cleared and double-stained. Additionally, vertebral counts were verified and supplemented through X-ray radiography performed on two specimens (voucher nos. ECSFRI 17262 and 25137). Morphometric data are presented as percentages of standard length (SL) and head length (HL). To enhance the visibility of minute anatomical structures, specimens were temporarily stained with Cyanine Blue 5R following the protocols of Akihito et al. [35] and Saruwatari et al. [36]. Vertebral counts were obtained using dual approaches: X-ray radiography and cleared-and-stained skeletal preparations. The clearing and staining procedure adhered to the methods of Dingerkus and Uhler [37] as modified by Taylor and Van Dyke [38].

## 3. Results

### 3.1. Morphological Description

Description. Based on 15 specimen, 57.70–83.99 mm SL. Proportional measurements and counts are given in Table 1.

**Figure 2:**
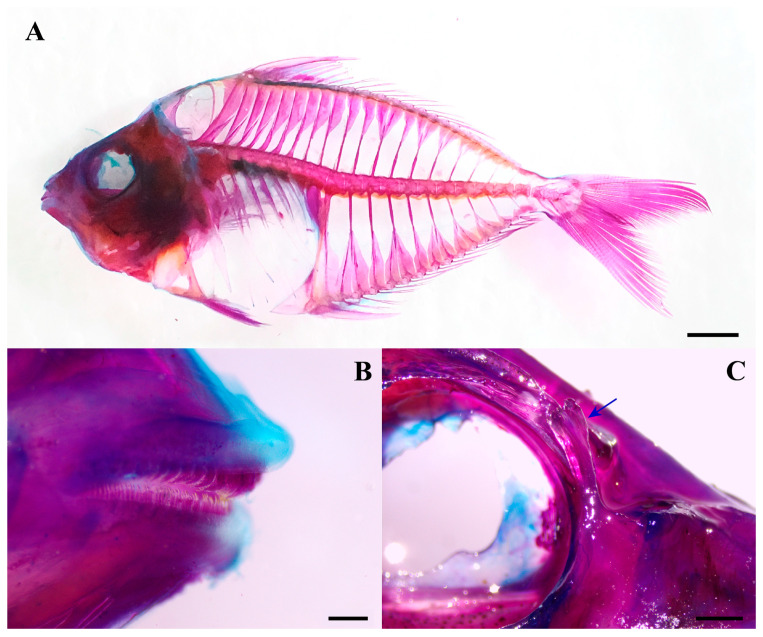
Dentition and preorbital spine morphology in *N. longicornis* based on cleared and stained specimens. (**A**) Left lateral view (ECSFRI 25137). Scale bar = 10 mm. (**B**) Teeth, right lateral view (ECSFRI 25140). Scale bar = 0.5 mm. (**C**) Preorbital spine, partial right lateral view (ECSFRI 25140). Scale bar = 1 mm. Arrows indicate the position of the preorbital spine. Photo by Ning Ya-Yang.

Body oval, laterally compressed; depth at dorsal-fin origin 43.9 (42–46)% SL. Dorsal and ventral profiles equally convex; occipital region slightly concave; head profile triangular. Snout black, pointed, with protrusible oral tube; length 29.6 (23–35)% HL, less than eye diameter (34.5% HL). Lower jaw arched when closed. Caudal peduncle short, narrow; length 9.9 (8–12)% SL, depth 6.1 (6–7)% SL. Vertebrae 24. Ninth (last precaudal) and tenth (first caudal) vertebrae transitional. Ninth vertebra with haemal arches and ribs; transverse processes elongated laterally. Tenth vertebra haemal arch broad, thin-plated, incurved bilaterally with medial notch distally (Figure 1C,F and Figure 2A). Teeth villiform (brush-like), short, slender, with incurved tips, arranged in 1–2 rows centrally on the premaxilla and dentary; palatine, vomer, and tongue edentulous (Figure 2B).

Dorsal-fin rays VIII,16; anal-fin rays III,14; pectoral-fin rays I,17; pelvic-fin rays I,5. Dorsal-fin origin posterior to pectoral and pelvic-fin origins, anterior to anal-fin origin. First dorsal-fin spine short, 8.7 (6–13)% HL; second spine longest, 72.0 (67–76)% HL; spines 3–4 with serrated anterior margins. Anal-fin origin opposite sixth dorsal-fin spine base. First anal-fin spine short, 12.5 (9–15)% HL; second spine long, 51.6 (42–59)% HL, tip reaching beyond fourth but not fifth soft-ray base when depressed; third spine with serrated anterior margin. Dorsal and anal spines with basal membranous sheaths, membranes connecting adjacent elements; fin bases posteriorly aligned vertically. Pectoral fins pointed, long, 67.7 (62–74)% HL; upper insertion anterior to lower, tip extending beyond anus. Pelvic fins small, subthoracic, shorter than pectorals; origin posterior to dorsal fin; spine with membranous sheath, free posteriorly; rays appressed ventrally when depressed. Caudal fin forked; lobes equal, tips rounded. Anus anterior to anal-fin origin, opposite third dorsal-fin spine base.

**Figure 3:**
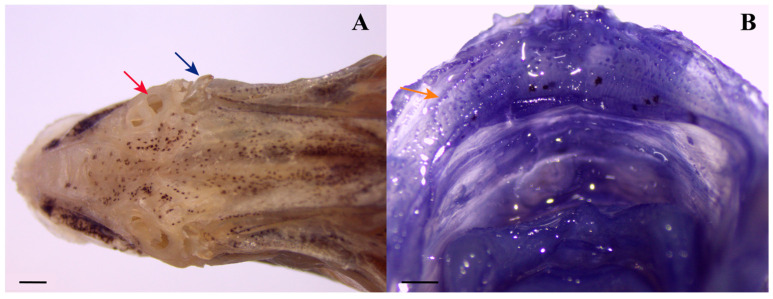
Preorbital spine and oral valve papillae of *N. longicornis* (ECSFRI 25136). (**A**) Dorsal view of preorbital spine (blue arrow) and nostrils (red arrows). Scale bar = 1 mm. (**B**) Minute papillae on upper oral valve (orange arrows). Scale bar = 0.5 mm. Photos by Ning-Ya Yang.

Mouth terminal, medium-sized; lips thin; gape horizontal at lower eye margin, slightly descending. Upper palate bearing numerous short, fine papillae along inner gingival margin (Figure 3B). Maxillary posterior limb exposed, attached to anterior cheek, extending beyond vertical through anterior eye margin. Eye large, diameter 34.5 (32–38)% HL; upper orbit convex, inner margin pigmented; ventral margin horizontal with upper pectoral-fin insertion. Adipose eyelid poorly developed. Preorbital spine present posterior to nostrils, branched distally, projecting laterally. Nostrils anterior to eye; anterior nostril small, circular; posterior nostril large, oval (Figure 3A).

Gill opening large, extending from postorbital region to posterior mandibular margin. Preopercle sickle-shaped, angle > 90°; inner margin arched; ventral arm smooth, dorsal arm serrated. Branchiostegal membrane attached to isthmus. Gill rakers: upper 5–6, lower 16–19, total 21–25. Cheek and breast asquamate, smooth; body otherwise covered with cycloid scales. Lateral line complete, slightly curved, its highest point below fifth dorsal-fin spine; descending from above opercle to above pectoral-fin base, arching posteriorly toward end of dorsal-fin base, then continuing horizontally along caudal peduncle; pored scales 50–59.

Coloration in life. Snout black. Head and body largely silvery-white; distinct black blotch on nape; minute melanophores scattered near mid-body axis. Dorsolateral surface with short, greyish or black worm-like markings or vertical bars. Lateral line yellow. Yellow patch between lower pectoral-fin insertion and anal-fin origin. Interradial membrane between third to seventh dorsal-fin spines yellow. Continuous black line along dorsal-fin base. Distal portions of anal-fin spines and rays yellow. Axillary region of pectoral fin yellow; pectoral-fin rays hyaline. Pelvic fins white. Distal tips of caudal-fin lobes light yellow (Figure 1A,D).

Coloration in preservative. Snout black; nape blotch faded. Dorsal body yellowish to light brown, with worm-like markings faded or yellowish; ventral body silvery-white. Pectoral-fin axilla black. Caudal-fin rays with black streaks; other fins hyaline (Figure 1B,E).

Distribution. *N. longicornis* was first recorded off the coast of Sri Racha, Gulf of Thailand [2], with subsequent reports from Malaysia [3], Vietnam [4], Myanmar [5] and now from the Beibu Gulf, South China Sea (present study).

### 3.2. Molecular Identification Based on 16S rRNA Gene

The phylogenetic analysis based on the 16S rRNA gene (Figure S1) successfully provided molecular identification for the examined *Nuchequula longicornis* specimens. The maximum likelihood (ML) tree showed that our specimens formed a clade with the reference sequence from Vietnam (LC770426), with a bootstrap support value of 79.4%. In contrast, the Bayesian inference (BI) tree presented a different topology: of this clade, one sequence (PX277132) formed a separate branch from the main cluster with a posterior probability of 0.875.

Beyond its utility and constraints in species identification, the 16S rRNA phylogeny also provided a broader perspective on the relationships within *Nuchequula*. Within *Nuchequula*, *N. blochii* formed an early-diverging lineage separate from the remaining species. The majority of *Nuchequula* species comprised a large clade in which *N. gerreoides* and *N. mannusella* grouped together with high ML bootstrap support (94.8%). In the Bayesian inference tree, however, a subclade of *N. mannusella* from Taiwan and Thailand was separated from *N. gerreoides*, though with low support (PP = 0.354). Additionally, *N. nuchalis* was paraphyletic with respect to a clade composed of *N. decora* and *N. longicornis*, wherein three *N. longicornis* sequences were nested within *N. decora*.

### 3.3. Characterization of the Complete Mitochondrial Genome

**Figure 4:**
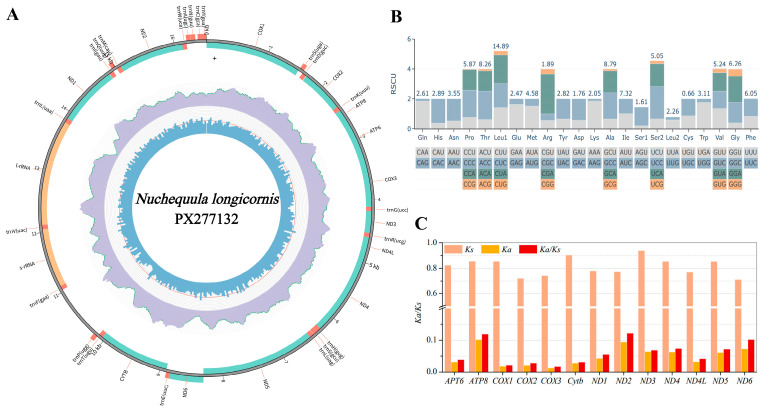
Mitogenomic characteristics of *N. longicornis* (GenBank accession no. PX277132). (**A**) Complete mitogenome map; innermost circles show GC content; purple ring indicate sequence coverage depth; outermost circle shows gene arrangement (green: PCGs; orange: rRNAs; red: tRNAs). (**B**) Relative synonymous codon usage (RSCU) across all protein-coding genes. (**C**) Ka, Ks, and Ka/Ks values for each PCG from nine Leiognathidae mitogenomes.

The complete mitogenome of *N. longicornis* (GenBank: PX277132) was a 16,514 bp circular molecule (Figure 4A; Table S2), containing the typical 37 mitochondrial genes and one control region. Except for the *ND6* gene and eight tRNAs encoded on the light strand, all other genes were located on the heavy strand, consistent with the gene arrangement observed in other Leiognathidae species. The genome exhibited a distinct AT bias (54.8% AT content; AT-skew = 0.090, GC-skew = −0.335; Table S2), with the highest AT content at the third codon position (58.6%; Table S5). The 13 protein-coding genes (total length 11,418 bp) used standard ATG initiation codons except for *COX1* (GTG), and seven genes terminated in incomplete stop codons (T/TA), presumed to be completed by polyadenylation (Table S4). Codon usage analysis indicated leucine (14.87%) as the most frequent amino acid and cysteine (0.16%) the least (Table S6; Figure 4B). To provide a genomic context within the genus, we compared the complete mitogenome of *N. longicornis* with that of its congener *Nuchequula nuchalis* (GenBank: AB355911). The two genomes shared an overall nucleotide sequence identity of 93.5%, a level typical for congeneric fish species. Evolutionary analysis revealed that all PCGs were under strong purifying selection (Ka/Ks < 1), with the highest ratio in *ND2* (0.1225) and the lowest in *COX3* (0.0170; Figure 4C). The 22 tRNAs (67–74 bp) all adopted typical cloverleaf secondary structures except *trnS1*, which lacked the DHU arm (Figure S2); 14 tRNAs were encoded on the heavy strand and 8 on the light strand (Table S4). The 12S and 16S rRNAs measured 948 bp and 1698 bp, respectively, and were located between *trnF* and *trnL* separated by *trnV* (Table S5). The rRNA region showed an AT content of 53.1% with a positive AT-skew (0.281). The genome contained six overlapping regions (longest 10 bp between *ATP8* and *ATP6*) and nine intergenic spacers (longest 36 bp between *trnN* and *trnC*; Table S4).

### 3.4. Phylogenetic Reconstruction

**Figure 5:**
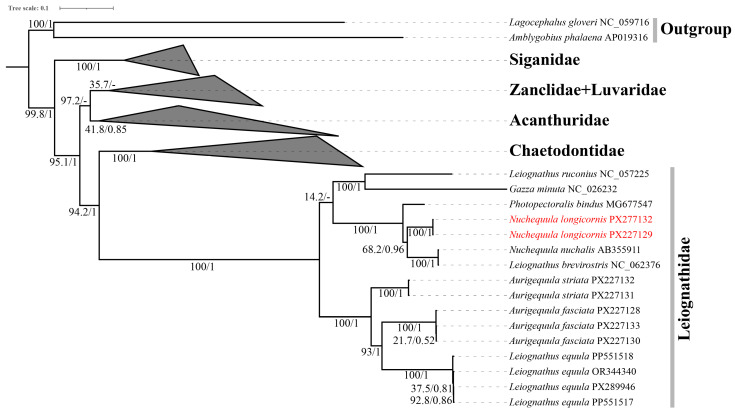
Phylogenetic position of Leiognathidae inferred from the nucleotide sequences of 13 mitochondrial protein-coding genes (without codon partitioning) analyzed by Bayesian inference (BI) and maximum likelihood (ML). Numbers above branches indicate ML bootstrap and Bayesian posterior probabilities, respectively; “-” indicates absent from maximum clade credibility tree. The newly sequenced specimens in this study are highlighted in red.

The mitogenome-based phylogeny was reconstructed from the nucleotide sequences of the 13 protein-coding genes, aligned into a concatenated dataset of 11,376 bp and analyzed without codon partitioning (Figure 5). In the resulting topology, *Nuchequula longicornis* was positioned as the sister. This specific relationship resulted in a topology where the other included congener, *N. nuchalis*, along with the sequence ‘*Leiognathus brevirostris*’ (which should be reassigned to the genus *Nuchequula* [18]). The combined (*Photopectoralis bindus* + *Nuchequula*) clade was sister to a clade comprising *Gazza minuta* and *Leiognathus ruconius* albeit with low ML bootstrap (14.2%). As noted in the Methods, differences in nodal support between ML and BI analyses can arise from their distinct statistical foundations. Consequently, in the following discussion, greater emphasis is placed on phylogenetic relationships that receive consistent and strong support from both inference methods.

Within the subfamily Leiognathinae, the specimens of *Aurigequula striata* formed a distinct branch. Nested within this branch was a well-supported clade comprising multiple specimens of *Leiognathus equula* and *Aurigequula fasciata*, highlighting the close phylogenetic association between these genera.

Overall, the mitogenome phylogeny provided stronger nodal support for most relationships compared to the 16S rRNA gene tree. It robustly supported the monophyly of *Nuchequula* and clarified the precise sister-group relationship between this genus and *Photopectoralis bindus*. The broader structure of the Leiognathidae family was consistent with the topology suggested by the 16S data, although the exact branching pattern among the major Gazzinae lineages remains uncertain due to low statistical support. Furthermore, beyond the internal structure of Leiognathidae, the broader phylogenetic context was clarified. The family Leiognathidae as a whole was robustly recovered as a monophyletic sister group to the family Chaetodontidae. This combined clade was, in turn, sister to a clade comprising the families Acanthuridae, Luvaridae, and Zanclidae. This topology, which is congruent with recent mitogenomic analyses [18], strongly supporting a revised systematic placement of Leiognathidae within the order Chaetodontiformes. A complete, uncollapsed version of the phylogenetic tree is provided as Supplementary Figure S3.

## 4. Discussion

### 4.1. Morphological Comparison and Interpretation of Variation

Our morphological comparison is based on the published description, as direct examination of the type series or topotypic specimens was not feasible for this study. Within this context, the specimens of *N. longicornis* from the Beibu Gulf agreed with the type series from the Gulf of Thailand in general morphometric and meristic characters (Table 1). A notable discrepancy was observed in the length of the second dorsal-fin spine. In our material, this measurement did not reach the maximum value of 102% SL reported in the type series, with a mean difference exceeding 15% of SL. Initial examination revealed clear evidence of spine breakage in three individuals; however, a thorough re-examination indicated that all 15 specimens exhibited varying degrees of overt or suspected damage to the second and third dorsal spines. This near-ubiquitous condition leads us to conclude that the observed disparity is most parsimoniously explained by the fragility and frequent breakage of these elongated spines during capture and preservation, a well-documented taphonomic challenge in Leiognathidae.

This interpretation is consistent with difficulties noted in the literature. The original description [2] characterized the second dorsal-fin spine as extremely elongated, yet subsequent studies [5,39] rarely document specimens unequivocally matching this trait; only the holotype unambiguously exhibits it. This suggests the characterization itself was based on a few, possibly intact, specimens and that breakage commonly obscures the true morphology in collected material. Furthermore, within the genus *Nuchequula*, the second dorsal spine is reported as highly variable, and the values for *N. longicornis* itself were derived from a very small sample [2], potentially not representative of its full post-collection variation.

To further investigate the nature of other minor morphometric variations (Table 1), we conducted exploratory analyses on our sample. Given the limited sample size, robust allometric modeling was not feasible. However, we examined the scaling relationships of these proportional measurements against standard length (Figure S4). No strong or consistent allometric trends were detected that would explain the observed mean differences from the type series as a function of body size. The variation within our sample appears scattered and within the range of ordinary intraspecific fluctuation. This supports the interpretation that these subtle discrepancies are more likely attributable to a combination of limited sampling, potential minor inter-observer measurement variation, and natural individual variation, rather than to directional allometric change or a systematic geographic divergence. Thus, we attribute the shorter spine lengths in our material not to true morphological divergence, but to post-collection breakage, a common taphonomic artifact in leiognathids. The comparison underscores that absolute measurements of such delicate structures in museum specimens should be interpreted with caution, and highlights the value of examining fresh material or using non-metric characters for reliable comparisons.

### 4.2. Phylogenetic Position and Implications

The mitogenome-based phylogeny robustly resolved the position of *N. longicornis* (Figure 5). Notably, it formed a sister-group relationship with *Photopectoralis bindus*, a topology distinct from its morphological congeners within *Nuchequula*. This placement suggests that the elongated second dorsal-fin spine may be homoplastic or represent a retained ancestral trait, highlighting the potential for morphological convergence in this character. Furthermore, the recovery of *N. longicornis* as sister to *Photopectoralis* bindus renders the genus *Nuchequula* non-monophyletic in our mitogenomic analysis. This result, which contrasts with multi-locus studies, highlights the uncertainty and potential for conflict when using limited mitochondrial data alone, and underscores the need for a robust molecular framework to guide future generic revisions. The low support for some internal nodes within the Gazzinae clade underscores the rapid diversification or persistent gene flow that may characterize this group’s evolutionary history. However, the novel sister-group relationship between *N. longicornis* and *Photopectoralis bindus* warrants careful consideration given certain limitations of the dataset.

In our mitogenomic phylogeny, *N. longicornis* was recovered as a sister lineage to *Photopectoralis bindus* (Figure 5). While this topology is robust within our dataset, its taxonomic implications must be interpreted with caution due to inherent limitations. First, the current representation of leiognathid mitogenomes in public databases is limited, which may result in a simplified or unstable topology that does not reflect the full complexity of family-level relationships. Second, this result contrasts with a recent multi-locus study (16S, COI, ND5) that recovered *Nuchequula* and *Karalla* as mutually monophyletic sister genera [40]. Such incongruence can arise from analytical factors like incomplete lineage sorting, long-branch attraction, or the different evolutionary histories captured by single versus concatenated markers. Therefore, while the *N. longicornis-Photopectoralis* sister relationship presents an interesting hypothesis—potentially indicating morphological convergence or ancestral trait retention—we emphasize that it should not be viewed as a conclusive redefinition of generic boundaries. Rather, it underscores the necessity for future studies to test this relationship with expanded taxon sampling and, crucially, independent data from nuclear genomic loci to obtain a fully resolved and robust phylogeny of Leiognathidae.

### 4.3. Biogeographic and Ecological Context

*Nuchequula longicornis* has been recorded at sizes up to 79 mm SL (FRLM 32385) and 80 mm SL in Myanmar [5], with a maximum reported size of 100 mm SL in Vietnam [4]. In the present study, the largest specimen measured 83.99 mm SL (ECSFRI 25135). The species is usually found over muddy bottoms in coastal inshore waters [4]. Its teeth form a specialized scraping apparatus, consistent with a diet of microscopic organisms and detritus. The Beibu Gulf collection site lies within a contiguous biogeographic region encompassing the known range of *N. longicornis* in Vietnam and the Gulf of Thailand, sharing comparable subtropical coastal environments with muddy substrates. The seasonal circulation patterns of the South China Sea, particularly the well-mixed cyclonic gyre within the Beibu Gulf as revealed by recent observations [41], may facilitate larval dispersal and gene flow among these areas. While empirical confirmation of population connectivity would require a multi-marker approach integrating tools such as otolith chemistry and genetic data [42], the potential for ongoing demographic connectivity provides a parsimonious explanation for the observed morphological and genetic homogeneity. Therefore, the minor morphological differences recorded are best interpreted as intraspecific variation within a potentially connected metapopulation.

### 4.4. Methodological Insights and Future Directions

Molecular phylogenetic analyses provided insights complementary to morphology but also highlighted methodological constraints. The topological inconsistencies and limited resolution at both genus and species levels in our 16S rRNA phylogeny (Figure S1) exemplify the challenges of using a single, conserved marker in taxonomically complex groups like *Nuchequula* [1,7,15]. Importantly, the more robust mitogenomic phylogeny (Figure 5) not only clarified the specific position of *N. longicornis* (as detailed above) but also corroborated broader, family-level systematic relationships. Our analysis recovered Leiognathidae as a monophyletic sister group to Chaetodontidae, with this clade being sister to Acanthuridae, Luvaridae, and Zanclidae. This topology aligns with recent mitogenomic studies [39] and strongly supports the placement of Leiognathidae within the order Chaetodontiformes. These results underscore that while mitochondrial genomes provide a powerful framework, the persistent uncertainty at some nodes highlights the need for future studies to integrate multiple nuclear loci for a fully resolved phylogeny of Leiognathidae.

In summary, both single mitochondrial genes and complete mitogenomes, as sole markers, exhibit inherent limitations in resolving phylogenetic relationships at various taxonomic levels. To establish a more robust and reliable phylogenetic framework for Leiognathidae—and to critically test hypotheses concerning genus monophyly and species boundaries—future studies should integrate multiple independent nuclear loci, as demonstrated in previous research [11].

## 5. Conclusions

This study reports the first record of *Nuchequula longicornis* from Chinese waters (Beibu Gulf) and presents its complete mitochondrial genome. Morphological examination showed overall congruence with the established description, with the most notable deviation in the second dorsal-fin spine length attributed to preservation artifact. Molecular data from the 16S rRNA gene provided consistent supporting evidence. Together, this integrative approach provides the strongest available evidence supporting the identification of this population as *N. longicornis*. Furthermore, we present the first complete mitochondrial genome for this species, which exhibits typical architecture and strong purifying selection, providing an essential genomic resource. Phylogenomic analysis based on this mitogenome robustly resolved *N. longicornis* as a sister lineage to *Photopectoralis bindus* within our dataset. It should be noted that this specific topology differs from the prevailing view of *Nuchequula* monophyly supported by recent multi-locus studies, likely reflecting the inherent limitations of a single genomic locus or current taxon sampling. This finding underscores the need for future studies to integrate multiple nuclear markers with comprehensive morphological data to test phylogenetic hypotheses and resolve the remaining taxonomic complexities within Leiognathidae. Finally, to conclusively validate the identity of the Beibu Gulf population and to rule out geographic variation, sequencing topotypic specimens of *N. longicornis* from the Gulf of Thailand would be highly valuable.

## Figures and Tables

**Table 1:** Comparison of morphometric and meristic characters between *N. longicornis* specimens examined in this study and the type material *.

Counts and Measurements	This Study	Kimura et al. [2]	
		Holotype	Paratypes
Standard length (mm)	57.70–83.99 (75.66, 15)	63	56–79 (66.2, 21)
Counts			
Dorsal fin rays	VIII, 16 (16, 15)	VIII, 16	VIII, 16–17 (16.0, 21)
Anal fin rays	III, 14 (14, 15)	III, 14	III, 14 (21)
Pectoral fin rays	16–19 (17, 15)	18	18–19 (18.2, 21)
Lateral line scales	50–59 (56, 15)	58	54–58 (55.7, 21)
Scale rows above lateral line	11–16 (13, 15)	14	10–15 (13.0, 17)
Scale rows below lateral line	26–33 (29, 15)	34	27–34 (30.0, 17)
Gill rakers on upper arch	5–6 (6, 3)	6	5–6 (5.8, 20)
Gill rakers on lower arch	16–19 (18, 3)	17	16–17 (16.8, 20)
Total gill rakers	21–25 (24, 3)	23	22–23 (22.5, 20)
Measurements			
As % of standard length			
Head length	28–32 (30.6, 15)	32	30–34 (32.3, 21)
Body depth	42–46 (43.9, 15)	47	44–49 (47.1, 21)
Caudal peduncle length	8–12 (9.9, 15)	11	8.6–12 (10.4, 21)
Caudal peduncle depth	6–7 (6.1, 15)	6.2	5.1–6.1 (5.7, 21)
Dorsal fin base length	53–57 (54.3, 15)	57	54–60 (56.1, 21)
Anal fin base length	42–45 (43.1, 15)	46	42–48 (44.8, 21)
Predorsal length	33–39 (36.6, 15)	45	45–49 (46.5, 21)
Pectoral-fin insertion to pelvic fin insertion	19–22 (20.8, 15)	21	20–23 (21.5, 21)
Pelvic-fin insertion to anal fin origin	16–20 (17.7, 15)	16	15–22 (17.6, 21)
Pelvic-fin insertion to anus center	6–9 (7.8, 15)		
Snout to pectoral-fin insertion	29–34 (31.9, 15)	34	33–36 (34.4, 21)
Snout to pelvic-fin insertion	36–39 (37.7, 15)	40	38–43 (40.4, 21)
Snout to anal-fin insertion	52–56 (53.9, 15)	56	54–59 (57.0, 21)
As % of head length			
Snout length	23–35 (29.6, 15)	35	32–38 (34.3, 21)
Upper jaw length	42–48 (44.5, 15)	42	40–47 (43.9, 21)
Length of posterior limb of maxilla	19–25 (21.3, 15)	24	17–27 (23.4, 21)
Interorbital width	28–32 (30.2, 15)	29	26–32 (29.1, 21)
Eye diameter/Orbit diameter	32–38 (34.5, 15)	36	32–37 (34.9, 21)
First dorsal fin spine length	6–13 (8.7, 15)	12	9.6–15 (12.3, 15)
Second dorsal fin spine length	67–76 (72.0, 12)	102	82–91 (86.5, 3)
Third dorsal fin spine length	41–67 (52.8, 15)	Damaged	65–71 (68.0, 2)
First anal fin spine length	9–15 (12.5, 15)	15	11–17 (14.8, 15)
Second anal fin spine length	42–59 (51.6, 15)	63	50–72 (60.1, 18)
Third anal fin spine length	31–44 (37.3, 12)	Damaged	47–50 (48.7, 28)
Pectoral fin length	62–74 (67.7, 15)	68	56–71 (63.0, 20)
Pelvic fin spine length	31–40 (35.0, 15)	39	32–39 (36.2, 19)

* For meristic counts in the present study, data are presented as range (median, n); for morphometric measurements, as range (mean, n). The values from Kimura et al. [2] are presented as originally published (mean for both counts and measurements).

## Data Availability

All of the data that support the findings of this study are available in the main text or Supplementary Information.

## Supplementary Materials

The following supporting information can be downloaded at: https://www.mdpi.com/article/10.3390/biology15030260/s1, Figure S1: Phylogenetic tree of Leiognathidae based on 16S rRNA gene sequences, analyzed by Bayesian inference (BI) and maximum likelihood (ML). The tree includes *Nuchequula longicornis* sequences from this study (in red) and 99 reference sequences from GenBank. Numbers above branches indicate ML bootstrap support values and Bayesian posterior probabilities, respectively; a dash (-) indicates nodes absent from the maximum clade credibility tree. This tree provides the molecular validation context discussed in Section 3.2; Figure S2: Secondary structure of the 22 tRNA genes of the mitochondrial genome of *N. longicornis*; Figure S3: Complete phylogenetic tree of Leiognathidae inferred from the analysis of 13 mitochondrial protein-coding genes, showing all taxa included in the analysis. Branches collapsed in the main text (Figure 5) for clarity are fully expanded here; Figure S4: Scatter plots showing the relationship between standard length (SL) and two proportional morphometric traits in the examined *Nuchequula longicornis* specimens. The lack of a clear trend supports the absence of strong allometric scaling within this sample; Table S1: 16S rRNA sequences used in the phylogenetic analysis; Table S2: Mitochondrial sequences used in the phylogenetic analysis as shown in this study; Table S3: Best partitioning schemes and models based on different datasets for Bayesian inference (BI) and maximum likelihood (ML) analysis; Table S4: Information on each gene fragment of *N. longicornis*; Table S5: Base composition of the *N. longicornis* mitochondrial genome.; Table S6: Codon number and RSCU of N. longicornis mitochondrial PCGs. References [7,10,18,40,43,44,45,46,47,48,49,50,51,52,53,54,55,56,57,58,59,60,61,62,63,64,65,66,67,68,69,70] are cited in the Supplementary Materials.
