# Supplementary material for: Integrative Taxonomy of Nuchequula longicornis (Teleostei: Leiognathidae) from Chinese Waters: Morphological Analysis, Mitogenomic Characterization, and Phylogenetic Implications
Source: Biology (Basel). 2026 Jan 30;15(3):260. doi: 10.3390/biology15030260 (PMC12897343; doi:10.3390/biology15030260)
Supplement: Supplementary file 1 [file biology-15-00260-s001.zip › Figure S1.pdf]

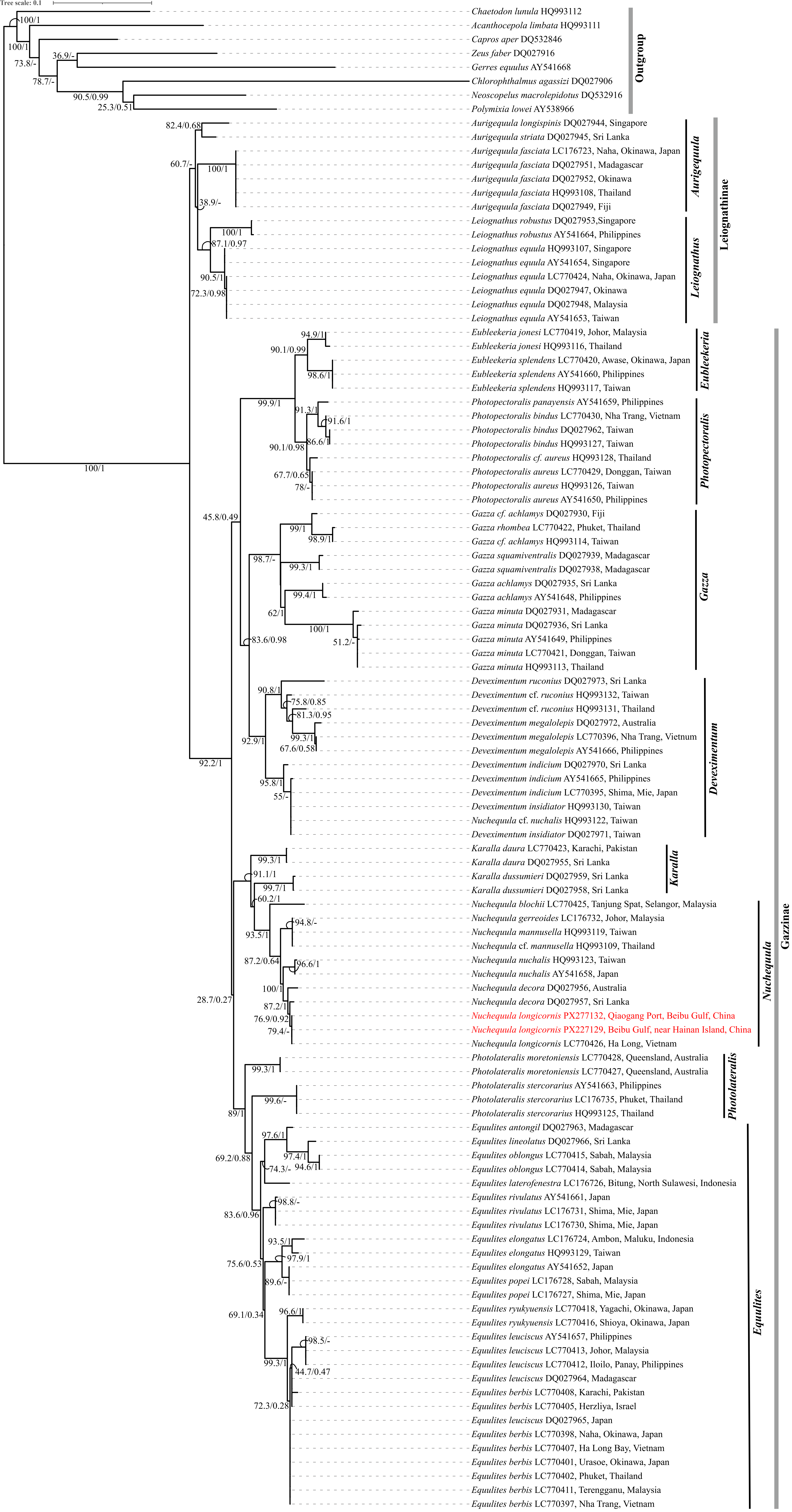

Figure S1 Phylogenetic tree of Leiognathidae based on 16S rRNA gene sequences, analyzed by Bayesian in-ference (BI) and maximum likelihood (ML). The tree includes *Nuchequula longicornis* sequences from this study (in red) and 99 reference sequences from GenBank. Numbers above branches indicate ML bootstrap support values and Bayesian posterior probabilities, respectively; a dash (-) indicates nodes absent from the maximum clade credibility tree. This tree provides the molecular validation context discussed in section 3.2.
