# Supplementary material for: Integrative Taxonomy of Nuchequula longicornis (Teleostei: Leiognathidae) from Chinese Waters: Morphological Analysis, Mitogenomic Characterization, and Phylogenetic Implications
Source: Biology (Basel). 2026 Jan 30;15(3):260. doi: 10.3390/biology15030260 (PMC12897343; doi:10.3390/biology15030260)
Supplement: Supplementary file 1 [file biology-15-00260-s001.zip › Figure S3. Complete phylogenetic tree.pdf]

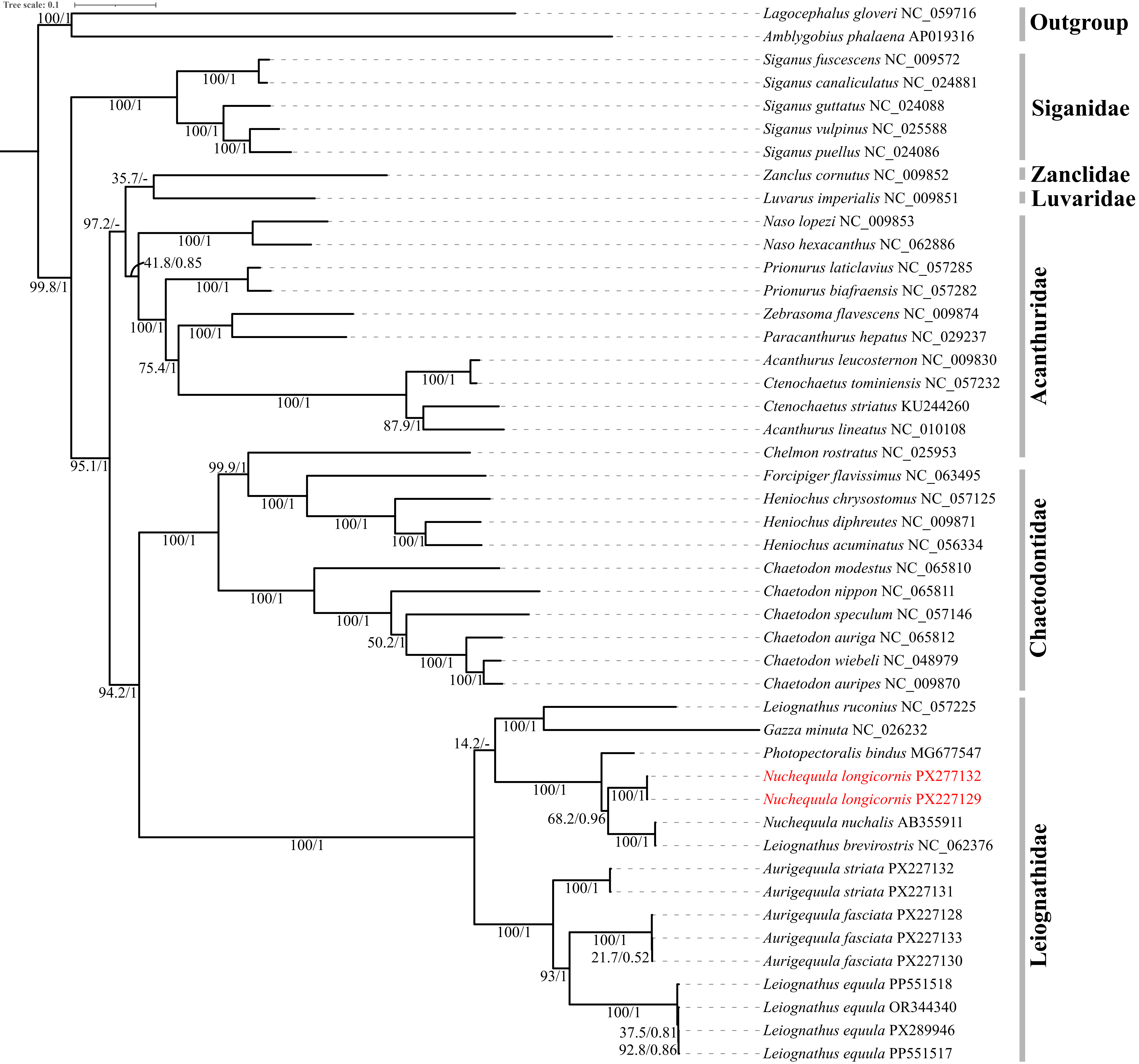

Figure S3 Complete phylogenetic tree of Leiognathidae inferred from the analysis of 13 mitochondrial protein-coding genes, showing all taxa included in the analysis. Branches collapsed in the main text (Figure 5) for clarity are fully expanded here.
