# Supplementary material for: Integrative Taxonomy of Nuchequula longicornis (Teleostei: Leiognathidae) from Chinese Waters: Morphological Analysis, Mitogenomic Characterization, and Phylogenetic Implications
Source: Biology (Basel). 2026 Jan 30;15(3):260. doi: 10.3390/biology15030260 (PMC12897343; doi:10.3390/biology15030260)
Supplement: Supplementary file 1 [file biology-15-00260-s001.zip › Figure S4. Scatter plots.pdf]

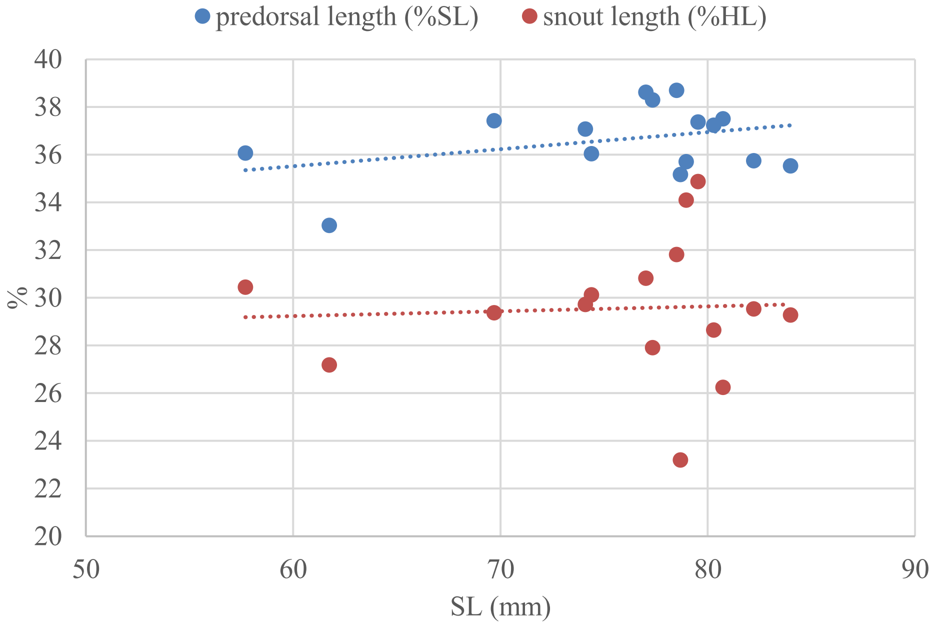

Figure S4 Scatter plots showing the relationship between standard length (SL) and two proportional morphometric traits in the examined *Nuchequula longicornis* specimens. The lack of a clear trend supports the absence of strong allometric scaling within this sample.
