# Supplementary material for: Integrative Taxonomy of Nuchequula longicornis (Teleostei: Leiognathidae) from Chinese Waters: Morphological Analysis, Mitogenomic Characterization, and Phylogenetic Implications
Source: Biology (Basel). 2026 Jan 30;15(3):260. doi: 10.3390/biology15030260 (PMC12897343; doi:10.3390/biology15030260)
Supplement: Supplementary file 1 [file biology-15-00260-s001.zip › Table S1-S6. of Nuchequula longicornis.pdf]

Table S1. 16S rRNA sequences used in the phylogenetic analysis.

Species	Accession no.	Voucher	Locality	References
**Leiognathidae**				
Aurigequula fasciata	DQ027949	KU 4649	Fiji	[1]
Aurigequula fasciata	DQ027951	JSS 28-2003 / LS 55	Madagascar	[1,2]
Aurigequula fasciata	DQ027952	FRLM 28999 / OKI-LF-1	Okinawa	[1]
Aurigequula fasciata	HQ993108	AMNH 240597	Thailand	[2]
Aurigequula fasciata	LC176723	FRLM 42591	Naha, Okinawa, Japan	[3]
Aurigequula longispinis	DQ027944	UMMZ 240361	Singapore	[1,2]
Aurigequula striata	DQ027945	LEI 2 SL	Sri Lanka	[1,2]
Deveximentum cf. ruconius	HQ993131	AMNH 240594	Thailand	[2]
Deveximentum cf. ruconius	HQ993132	AMNH 239260	Taiwan	[2]
Deveximentum indicium	DQ027970	LEI 12 SL	Sri Lanka	[1,2,4]
Deveximentum indicium	AY541665		Philippines	[4,5]
Deveximentum indicium	LC770395	FRLM 46528	Shima, Mie, Japan	[3]
Deveximentum insidiator	DQ027971	WLS 32 Tai	Taiwan	[1,2]
Deveximentum insidiator	HQ993130	AMNH 239272	Taiwan	[2]
Deveximentum megalolepis	AY541666		Philippines	[1,2,4]
Deveximentum megalolepis	DQ027972	WI-02-11	Australia	[1,2]
Deveximentum megalolepis	LC770396	FRLM 47670	Nha Trang, Vietnum	[3]
Deveximentum ruconius	DQ027973	LEI 14 SL	Sri Lanka	[1,2]
Equulites antongil	DQ027963	AMNH 119981	Madagascar	[1,2]
Equulites berbis	LC770397	FRLM 31455	Nha Trang, Vietnam	[3]
Equulites berbis	LC770398	FRLM 42588	Naha, Okinawa, Japan	[3]
Equulites berbis	LC770401	FRLM 43341	Urasoe, Okinawa, Japan	[3]
Equulites berbis	LC770402	FRLM 47106	Phuket, Thailand	[3]
Equulites berbis	LC770405	FRLM 48239	Herzliya, Israel	[3]
Equulites berbis	LC770407	FRLM 49665	Ha Long Bay, Vietnam	[3]
Equulites berbis	LC770408	FRLM 49836	Karachi, Pakistan	[3]
Equulites berbis	LC770411	KAUM–I. 16861	Terengganu, Malaysia	[3]
Equulites elongatus	AY541652	AMNH 122175	Japan	[1,2,4]
Equulites elongatus	HQ993129	AMNH Uncat.	Taiwan	[2]
Equulites elongatus	LC176724	FRLM 35775	Ambon, Maluku, Indonesia	[3]
Equulites laterofenestra	LC176726	FRLM 39985	Bitung, North Sulawesi, Indonesia	[3]
Equulites leuciscus	AY541657	AMNH 122173	Philippines	[1,2]
Equulites leuciscus	DQ027964	AMNH 120336	Madagascar	[1,2]
Equulites leuciscus	DQ027965	OKI-LL-1	Japan	[1,2]
Equulites leuciscus	LC770412	FRLM 32730	Iloilo, Panay, Philippines	[3]

Species	Accession no.	Voucher	Locality	References
Equulites leuciscus	LC770413	FRLM 47197	Johor, Malaysia	[3]
Equulites lineolatus	DQ027966	LEI 16 SL	Sri Lanka	[1,2]
Equulites oblongus	LC770414	KAUM–I. 12531	Sabah, Malaysia	[3]
Equulites oblongus	LC770415	KAUM–I. 12575	Sabah, Malaysia	[3]
Equulites popei	LC176726	FRLM 36591	Owase, Mie, Japan	[3]
Equulites popei	LC176727	FRLM 45856	Shima, Mie, Japan	[3]
Equulites popei	LC176728	KAUM–I. 12011	Sabah, Malaysia	[3]
Equulites rivulatus	AY541661		Japan	[1,2,4]
Equulites rivulatus	LC176730	FRLM 34602	Shima, Mie, Japan	[3]
Equulites rivulatus	LC176731	FRLM 34605	Shima, Mie, Japan	[3]
Equulites ryukyuensis	LC770416	FRLM 31846	Shioya, Okinawa, Japan	[3]
Equulites ryukyuensis	LC770418	FRLM 43321	Yagachi, Okinawa, Japan	[3]
Eubleekeria jonesi	HQ993116	AMNH 240596	Thailand	[2]
Eubleekeria jonesi	LC770419	FRLM 43934	Johor, Malaysia	[3]
Eubleekeria splendens	AY541660	AMNH 122187	Philippines	[1,2]
Eubleekeria splendens	HQ993117	AMNH 239273	Taiwan	[2]
Eubleekeria splendens	LC770420	FRLM 37744	Awase, Okinawa, Japan	[3]
Gazza achlamys	AY541648		Philippines	[1,2,4,5]
Gazza achlamys	DQ027935	LEI 1 SL	Sri Lanka	[1,2,4]
Gazza cf. achlamys	DQ027930	KU 4444	Fiji	[1,2]
Gazza cf. achlamys	HQ993114	AMNH 239279	Taiwan	[2]
Gazza minuta	DQ027931	AMNH 120340	Madagascar	[1,2]
Gazza minuta	DQ027936	LEI 6 SL	Sri Lanka	[1,2]
Gazza minuta	AY541649		Philippines	[1,5]
Gazza minuta	HQ993113	AMNH 240585	Thailand	[2]
Gazza minuta	LC770421	FRLM 47038	Donggan, Taiwan	[3]
Gazza rhombea	LC770422	FRLM 47075	Phuket, Thailand	[3]
Gazza squamiventralis	DQ027938	AMNH 120341	Madagascar	[1,2]
Gazza squamiventralis	DQ027939	AMNH 120342	Madagascar	[1,2]
Karalla daura	DQ027955	LEI 8 SL	Sri Lanka	[1,2]
Karalla daura	LC770423	FRLM 49845	Karachi, Pakistan	[3]
Karalla dussumieri	DQ027958	LEI 10 SL	Sri Lanka	[1,2]
Karalla dussumieri	DQ027959	AMNH 234763	Sri Lanka	[1,2]
Leiognathus equula	HQ993107	AMNH Uncat.	Singapore	[2]
Leiognathus equulus	AY541653	AMNH 122185	Taiwan	[1,2,4]
Leiognathus equulus	DQ027948	WLS 14 Tai	Malaysia	[1,2,4]
Leiognathus equulus	AY541654	UMMZ 238805	Singapore	[1,4]
Leiognathus equulus	DQ027947	OKI-LE-1	Okinawa	[1,4]

Species	Accession no.	Voucher	Locality	References
Leiognathus equulus	LC770424	FRLM 42596	Naha, Okinawa, Japan	[3]
Leiognathus robustus	AY541664	UMMZ 242144	Philippines	[1,2]
Leiognathus robustus	DQ027953	UMMZ 240360	Singapore	[1,2]
Nuchequula blochii	LC770425	FRLM 33651	Tanjung Spat, Selangor, Malaysia	[3]
Nuchequula cf. mannusella	HQ993109	AMNH 240599 (SI-41)	Thailand	[2]
Nuchequula cf. nuchalis	HQ993122	AMNH 239290	Taiwan	[2]
Nuchequula decora	DQ027957	AMNH 234765	Sri Lanka	[1,2]
Nuchequula decora	DQ027956	WI-02–04	Australia	[1,2,4]
Nuchequula gerreoides	LC176732	FRLM 43973	Johor, Malaysia	[3]
Nuchequula longicornis	PX227129	ECSFRI 17255	Beibu Gulf, near Hainan Island, China	This study
Nuchequula longicornis	PX277132	ECSFRI 25137	Qiaogang Port, Beibu Gulf, China	This study
Nuchequula longicornis	LC770426	FRLM 49482	Ha Long, Vietnam	[3]
Nuchequula mannusella	HQ993119	AMNH 238759	Taiwan	[2]
Nuchequula nuchalis	AY541658		Japan	[1,2]
Nuchequula nuchalis	HQ993123	AMNH 239165	Taiwan	[2]
Photolateralis moretoniensis	LC770427	CSIRO H 6940021	Queensland, Australia	[3]
Photolateralis moretoniensis	LC770428	CSIRO H 6940022	Queensland, Australia	[3]
Photolateralis stercorarius	AY541663	AMNH 122172	Philippines	[1,4]
Photolateralis stercorarius	HQ993125	AMNH 240588	Thailand	[2]
Photolateralis stercorarius	LC176735	FRLM 33704	Phuket, Thailand	[3]
Photopectoralis aureus	AY541650	AMNH 122186	Philippines	[1,2]
Photopectoralis aureus	HQ993126	ASIZ0801114	Taiwan	[2]
Photopectoralis aureus	LC770429	FRLM 47039	Donggan, Taiwan	[3]
Photopectoralis cf. aureus	HQ993128	AMNH 240591	Thailand	[2]
Photopectoralis bindus	LC770430	FRLM 47638	Nha Trang, Vietnam	[3]
Photopectoralis bindus	DQ027962	WLS 31 Tai	Taiwan	[1,2]
Photopectoralis bindus	HQ993127	AMNH 239275	Taiwan	[2]
Photopectoralis panayensis	AY541659	AMNH 122174	Philippines	[1,2]
**Outgroup**				
Acanthocepola limbata	HQ993111			[2,6]
Capros aper	DQ532846			[2,6]
Chaetodon lunula	HQ993112			[1,2]
Chlorophthalmus agassizi	DQ027906			[1,2]
Gerres equulus	AY541668			[1,2]
Neoscopelus macrolepidotus	DQ532916			[2,6]
Polymixia lowei	AY538966			[1,2]
Zeus faber	DQ027916			[1,2]

Table S2. Mitochondrial sequences used in the phylogenetic analysis as shown in this study.

Species	Size (bp)	Accession no.	A (%)	T (%)	C (%)	G (%)	A+T (%)	A+T skew	G+C skew	References
**Out group**										
Lagocephalus gloveri	16446	NC_059716	27.6	25.1	16.5	30.8	52.7	0.048	-0.302	[7,8]
Amblygobius phalaena	16622	AP019316	26.2	25.0	18.7	30.0	51.2	0.024	-0.231	[8,9]
**Siganidae**										
Siganus fuscescens	16491	NC_009572	28.1	25.4	16.8	29.8	53.5	0.050	-0.280	[8,10]
Siganus canaliculatus	16492	NC_024881	28.1	25.4	16.7	29.7	53.5	0.051	-0.281	[8,10,11]
Siganus guttatus	16505	NC_024088	29.3	25.7	15.7	29.3	55.0	0.064	-0.302	[8,10,12]
Siganus vulpinus	16505	NC_025588	28.9	25.5	16.0	29.6	54.4	0.064	-0.300	[8,10,13]
Siganus puellus	16504	NC_024086	28.6	25.6	16.3	29.5	54.2	0.056	-0.288	[8,10,14]
**Zanclidae**										
Zanclus cornutus	16521	NC_009852	26.4	26.3	17.6	29.7	52.7	0.002	-0.257	[8,15]
**Luvaridae**										
Luvarus imperialis	16497	NC_009851	26.8	25.5	17.6	30.1	52.3	0.025	-0.261	[8,15]
**Acanthuridae**										
Naso lopezi	16542	NC_009853	28.9	26.2	15.9	29.0	55.1	0.048	-0.290	[8,10,15,16]
Naso hexacanthus	16611	NC_062886	29.0	25.8	16.1	29.1	54.8	0.059	-0.287	[8,17]
Prionurus laticlavius	16531	NC_057285	29.4	26.3	15.4	28.8	55.7	0.057	-0.302	[8,16]
Prionurus biafraensis	16552	NC_057282	29.2	26.0	15.6	28.8	55.2	0.058	-0.298	[8,16]
Zebrasoma flavescens	16503	NC_009874	28.9	25.5	16.1	29.5	54.4	0.062	-0.295	[8,10,15,16]
Paracanthurus hepatus	16498	NC_029237	28.6	26.4	16.3	28.6	55.0	0.039	-0.274	[8,10,16]
Acanthurus leucosternon	16434	NC_009830	29.3	26.4	15.8	28.5	55.7	0.052	-0.287	[8,18]
Ctenochaetus tominiensis	16442	NC_057232	29.2	26.3	15.8	28.6	55.5	0.052	-0.287	[8,19]
Ctenochaetus striatus	17272	KU244260	29.4	26.5	15.8	28.3	55.9	0.051	-0.285	[8,16]
Acanthurus lineatus	16532	NC_010108	29.8	26.6	15.5	28.1	56.4	0.058	-0.289	[8,20]
**Leiognathidae**										
Leiognathus ruconius	16465	NC_057225	31.6	25.2	14.5	28.7	56.8	0.112	-0.327	[8,21]
Gazza minuta	16475	NC_026232	29.5	25.3	15.8	29.5	54.8	0.076	-0.303	[8]
Photopectoralis bindus	16517	MG677547	29.9	25.0	15.1	30.1	54.9	0.089	-0.333	[8,22]
Nuchequula nuchalis	15965	AB355911	29.8	25.3	15.2	29.7	55.1	0.080	-0.322	[8,23]
"Leiognathus brevirostris"	16465	NC_062376	29.6	25.5	15.3	29.6	55.1	0.074	-0.318	[8]
Nuchequula longicornis	16592	PX227129	29.9	25.0	15.0	30.0	54.9	0.089	-0.332	This study
Nuchequula longicornis	16514	PX277132	29.9	24.9	15.0	30.2	54.8	0.090	-0.335	This study
Aurigequula striata	16629	PX227131	30.7	24.0	14.6	30.6	54.7	0.123	-0.354	[24]
Aurigequula striata	16584	PX227132	30.8	24.0	14.6	30.7	54.8	0.124	-0.355	[24]
Aurigequula fasciata	16489	PX227130	30.5	23.9	15.3	30.3	54.4	0.121	-0.330	[24]
Aurigequula fasciata	16523	PX227133	30.5	24.0	15.3	30.2	54.5	0.120	-0.329	[24]

Species	Size (bp)	Accession no.	A (%)	T (%)	C (%)	G (%)	A+T (%)	A+T skew	G+C skew	References
Aurigequula fasciata	16537	PX227128	30.6	24.0	15.3	30.2	54.6	0.121	-0.329	[24]
Leiognathus equula	16400	PX289946	30.7	24.2	15.0	30.1	54.9	0.117	-0.334	[24]
Leiognathus equula	16398	OR344340	30.7	24.2	15.0	30.1	54.9	0.117	-0.334	[8]
Leiognathus equula	16399	PP551517	30.6	24.3	15.0	30.1	54.9	0.117	-0.333	[8]
Leiognathus equula	16395	PP551518	30.7	24.2	15.0	30.1	54.9	0.117	-0.333	[8]
**Chaetodontidae**										
Chelmon rostratus	16538	NC_025953	28.9	28.1	16.2	26.8	57.0	0.015	-0.246	[8,25]
Forcipiger flavissimus	16600	NC_063495	27.8	26.9	16.9	28.4	54.7	0.017	-0.254	[8]
Heniochus chrysostomus	16650	NC_057125	28.6	25.5	16.2	29.6	54.1	0.059	-0.292	[8,26]
Heniochus diphreutes	16772	NC_009871	29.2	25.8	15.6	29.4	55.0	0.061	-0.305	[8,15]
Heniochus acuminatus	16584	NC_056334	29.0	26.0	15.8	29.2	55.0	0.054	-0.299	[8,27]
Chaetodon modestus	16490	NC_065810	28.0	28.7	16.7	26.5	56.7	-0.012	-0.227	[8,28]
Chaetodon nippon	16507	NC_065811	27.5	27.9	16.8	27.8	55.4	-0.006	-0.248	[8,29]
Chaetodon speculum	16513	NC_057146	27.8	26.6	16.4	29.1	54.4	0.022	-0.278	[8,30]
Chaetodon auriga	16527	NC_065812	28.2	26.3	16.3	29.2	54.5	0.034	-0.284	[8,29]
Chaetodon wiebeli	16523	NC_048979	27.9	26.4	16.6	29.1	54.3	0.027	-0.274	[8,31]
Chaetodon auripes	16530	NC_009870	27.9	26.4	16.6	29.2	54.3	0.027	-0.276	[8,15]

References

1. Sparks, J.S.; Dunlap, P.V.; Smith, W.L. Evolution and Diversification of a Sexually Dimorphic Luminescent System in Ponyfishes (Teleostei: Leiognathidae), Including Diagnoses for Two New Genera. *Cladistics* **2005**, *21*, 305–327, doi:10.1111/j.1096-0031.2005.00067.x.
2. Chakrabarty, P.; Davis, M.P.; Smith, W.L.; Baldwin, Z.H.; Sparks, J.S. Is Sexual Selection Driving Diversification of the Bioluminescent Ponyfishes (Teleostei: Leiognathidae)?: Testing for Sexual Selection in Ponyfishes. *Molecular Ecology* **2011**, *20*, 2818–2834, doi:10.1111/j.1365-294X.2011.05112.x.
3. Suzuki, H.; Kimura, S. Taxonomic Revision of the Genus *Equulites* Fowler 1904 (Acanthuriformes: Leiognathidae). *Ichthyol Res* **2023**, *71*, 213–259, doi:10.1007/s10228-023-00935-z.
4. Ikejima, K.; Ishiguro, N.B.; Wada, M.; Kita-Tsukamoto, K.; Nishida, M. Molecular Phylogeny and Possible Scenario of Ponyfish (Perciformes:Leiognathidae) Evolution. *Mol Phylogenet Evol* **2004**, *31*, 904–909, doi:10.1016/j.ympev.2003.10.006.
5. Sparks, J.S.; Dunlap, Paul.V. A Clade of Non-Sexually Dimorphic Ponyfishes (Teleostei: Perciformes: Leiognathidae): Phylogeny, Taxonomy, and Description of a New Species. *American Museum Novitates* **2004**, *3459*, 1–21, doi:10.1206/0003-0082(2004)459%3C0001:ACONDP%3E2.0.CO;2.
6. Smith, W.L.; Wheeler, W.C. Polyphyly of the Mail-Cheeked Fishes (Teleostei: Scorpaeniformes): Evidence from Mitochondrial and Nuclear Sequence Data. *Mol Phylogenet Evol* **2004**, *32*, 627–646, doi:10.1016/j.ympev.2004.02.006.
7. Huang, X.; Shi, Y.; Shen, X.; Huang, D.; Wang, Y.; Chen, J.; Cai, Y. Characterization of the Complete Mitochondrial DNA Sequence of the *Lagocephalus Gloveri* (Tetraodontidae, Tetraodontiformes). *Mitochondrial DNA Part B* **2020**, *5*, 3665–3666, doi:10.1080/23802359.2020.1832933.
8. Chen, J.; Wang, X.; Zeng, S.; Tian, W.; Yang, D.; Ye, J.; Zhong, J.; Jiang, C. Morphometric and Phylogenetic Analysis of a Commercial Fish *Leiognathus Equula* (Teleostei, Leiognathidae). *ZK* **2024**, *1219*, 249–270, doi:10.3897/zookeys.1219.130546.
9. Hanahara, N.; Higashiji, T.; Shinzato, C.; Koyanagi, R.; Maeda, K. First Record of *Larsonella Pumilus* (Teleostei: Gobiidae) from Japan, with Phylogenetic Placement of the Genus *Larsonella*. *Zootaxa* **2019**, *4695*, 367–377, doi:10.11646/zootaxa.4695.4.4.
10. Huang, J.; Xue, X.N.; Wang, Q.; Hong, W.S.; Shen, K.-N.; Chen, S.X. The Complete Mitochondrial Genome of the Palette Surgeonfish, *Paracanthurus Hepatus*

(Perciformes: Acanthuridae). *Mitochondrial DNA A DNA Mapp Seq Anal* **2017**, *28*, 73–74, doi:10.3109/19401736.2015.1110804.
11. Zhou, L.; Xie, Z.; Zhang, Y. The Complete Mitochondrial Genome of the *Siganus Canaliculatus* (Perciformes: Siganidae). *Mitochondrial DNA Part A* **2016**, *27*, 1111–1112, doi:https://doi.org/10.3109/19401736.2014.933324.
12. Yan, S.; Wang, M.; Yang, C.; Yang, T. Complete Mitochondrial Genome of the Orange-Spotted Spinefoot *Siganus Guttatus* (Perciformes, Siganidae). *Mitochondrial DNA Part A* **2014**, *27*, 555–556, doi:10.3109/19401736.2014.905855.
13. Yan, S.; Wang, M.; Yang, T. Complete Mitogenome of the Foxface Rabbitfish Siganus Vulpinus (Perciformes, Siganidae): Indication of Potential Interbreeding in Rabbitfishes. *Mitochondrial DNA Part A* **2016**, *27*, 1906–1907, doi:10.3109/19401736.2014.971280.
14. Wang, M.; Yan, S.; Li, M.; Zhao, M.; Yang, T. Complete Mitochondrial Genome of the Masked Spinefoot Siganus Puellus (Perciformes, Siganidae). *Mitochondrial DNA* **2015**.
15. Yamanoue, Y.; Miya, M.; Matsuura, K.; Yagishita, N.; Mabuchi, K.; Sakai, H.; Katoh, M.; Nishida, M. Phylogenetic Position of Tetraodontiform Fishes within the Higher Teleosts: Bayesian Inferences Based on 44 Whole Mitochondrial Genome Sequences. *Molecular Phylogenetics and Evolution* **2007**, *45*, 89–101, doi:10.1016/j.ympev.2007.03.008.
16. Ludt, W.B.; Rocha, L.A.; Chakrabarty, P. The First Complete Mitochondrial Genomes of Sawtail Surgeonfishes (Acanthuridae: *Prionurus*). *Mitochondrial DNA Part B* **2020**, *5*, 212–213, doi:10.1080/23802359.2019.1699465.
17. Choi, H.; Kim, S.; Choi, H.; Youn, S. The Complete Mitochondrial Genome of Sleek Unicornfish, Naso Hexacanthus (Acanthuridae, Perciformes). *Mitochondrial DNA B Resour* **2023**, *8*, 274–275, doi:10.1080/23802359.2022.2160666.
18. Devadhasan, B.G.; Gnana, P.V.S.; Benes, V. Sequencing and Analysis of the Complete Mitochondrial Genome of the Surgeon Fish *Acanthurus Leucosternon BENNETT 1833 (Perciformes: Acanthuridae)* with Phylogenetic Consideration. *Mitochondrial DNA Part B* **2016**, *1*, 795–796, doi:10.1080/23802359.2016.1197068.
19. Jingni, C.; Haihao, H.; Jiantao, H.; Xiuying, Y. The Complete Mitochondrial Genome Sequence of *Ctenochaetus Tominiensis* (Actinopteri, Acanthuridae). *Mitochondrial DNA Part B* **2022**, *7*, 159–160, doi:10.1080/23802359.2021.2016078.
20. Devadhasan, B.G.; Gnana, P.V.S.; Benes, V. The Complete Mitochondrial Genome of the Surgeon Fish *Acanthurus Lineatus* Linnaeus 1758 (Perciformes: Acanthuridae). *Mitochondrial DNA Part B* **2016**, *1*, 748–749, doi:10.1080/23802359.2016.1197061.
21. Sui, Y.; Qin, B.; Song, X.; Sheng, W.; Zhang, B. Complete Mitochondrial Genome of the Deep Pugnose Ponyfish *Secutor Ruconius* (Perciformes: Leiognathidae) in the East China Sea. *Mitochondrial DNA Part B* **2019**, *4*, 3563–3564, doi:10.1080/23802359.2019.1670108.
22. Shi, W.; Wu, B.; Yu, H. The Complete Mitochondrial Genome Sequence of *Photopectoralis Bindus* (Perciformes: Leiognathidae). *Mitochondrial DNA Part B* **2018**, *3*, 71–72, doi:10.1080/23802359.2017.1422404.
23. Satoh, T.P.; Miya, M.; Mabuchi, K.; Nishida, M. Structure and Variation of the Mitochondrial Genome of Fishes. *BMC Genomics* **2016**, *17*, 719, doi:10.1186/s12864-016-3054-y.
24. Chen, J.-J.; Zhong, J.-S.; Sheng, Z.; Yang, D.-Y.; Liu, P.; Wang, X.-D.; Zhang, H.-Y.; Ye, J.-Q. A New Leiognathid Record from China with Complete Mitogenomes and Phylogenetic Insights of Two Aurigequula (Teleostei, Leiognathidae) Species. *ZooKeys* **2026**, in press.
25. Wang, L.-J.; You, F.; Wu, Z.-H. Complete Mitochondrial Genome of Copperband Butterflyfish *Chelmon Rostratus* (Teleostei, Perciformes, Chaetodontidae). *Mitochondrial DNA Part A* **2016**, *27*, 2141–2142, doi:10.3109/19401736.2014.982596.
26. Yu, W.; Yang, Y.; Qi, Z.; Shan, B.; Huang, X.; Liu, Y.; Lin, H.; Li, T.; Huang, Z.; Ma, Z.; et al. The Complete Mitochondrial Genome of *Heniochus Chrysostomus* (Perciformes, Chaetodontidae). *Mitochondrial DNA Part B* **2021**, *6*, 933–935, doi:10.1080/23802359.2021.1888335.
27. Jiang, F.; Yang, N.; Huang, H. Characterization and Phylogenetic Analysis of the Mitochondrial Genome Sequence of *Heniochus Acuminatus*. *Mitochondrial DNA Part B* **2022**, *7*, 1694–1695, doi:10.1080/23802359.2022.2049016.
28. Patil, M.P.; Kim, J.-O.; Lee, Y.-J.; Seo, Y.B.; Kim, J.-K.; Kim, G.-D. Complete Mitochondrial Genome of Brown-Banded Butterflyfish Chaetodon Modestus (Chaetodontiformes, Chaetodontidae) and Phylogenetic Analysis. *Mitochondrial DNA B Resour* **2022**, *7*, 2012–2014, doi:10.1080/23802359.2022.2148490.

29. Patil, M.P.; Kim, J.-O.; Lee, Y.-J.; Seo, Y.B.; Kim, J.-K.; Kim, G.-D. The Complete Mitochondrial Genome of Threadfin Butterflyfish, Chaetodon Auriga (Chaetodontiformes: Chaetodontidae) and Phylogenetic Analysis. *Mitochondrial DNA B Resour* **2022**, *7*, 1922–1924, doi:10.1080/23802359.2022.2136982.
30. Yang, Y.; Li, T.; Lin, H.; Huang, X.; Yu, W.; Huang, Z. The Complete Mitochondrial Genome of Chaetodon Speculum (Chaetodontiformes, Chaetodontidae). *Mitochondrial DNA B Resour* **2021**, *6*, 1290–1291, doi:10.1080/23802359.2021.1906170.
31. Yukai, Y.; Xiaolin, H.; Heizhao, L.; Tao, L.; Wei, Y.; Zhong, H. The Complete Mitochondrial Genome of Chaetodon Wiebeli (Chaetodontiformes, Chaetodontidae). *Mitochondrial DNA B Resour* **2019**, *4*, 3145–3146, doi:10.1080/23802359.2019.1667894.

Table S3 Best partitioning schemes and models based on different datasets for Bayesian inference (BI) and maximum likelihood (ML) analysis.

Dastaset	BI/ML	Subset partitions	Best model
Mitogenome	BI	P1: (atp6_mafft_trimAl_nad5_mafft_trimAl)	GTR+F+I+G4
		P2: (atp8_mafft_trimAl_nad4l_mafft_trimAl)	GTR+F+I+G4
		P3: (cox1_mafft_trimAl)	GTR+F+I+G4
		P4: (cox2_mafft_trimAl)	GTR+F+I+G4
		P5: (cox3_mafft_trimAl)	GTR+F+I+G4
		P6: (cytb_mafft_trimAl)	GTR+F+I+G4
		P7: (nad1_mafft_trimAl)	GTR+F+I+G4
		P8: (nad2_mafft_trimAl)	GTR+F+I+G4
		P9: (nad3_mafft_trimAl)	GTR+F+I+G4
		P10: (nad4_mafft_trimAl)	GTR+F+I+G4
		P11: (nad6_mafft_trimAl)	GTR+F+I+G4
	ML	P1: (atp6_mafft_trimAl_cytb_mafft_trimAl_nad1_mafft_trimAl_nad4_mafft_trimAl_nad5_mafft_trimAl)	TIM2+F+I+I+R4
		P2: (atp8_mafft_trimAl_nad3_mafft_trimAl_nad4l_mafft_trimAl)	TPM2u+F+I+G4
		P3: (cox1_mafft_trimAl)	TIM2+F+I+G4
		P4: (cox2_mafft_trimAl)	TIM2+F+I+G4
		P5: (cox3_mafft_trimAl)	TIM2+F+I+G4
		P6: (nad2_mafft_trimAl)	GTR+F+I+G4
		P7: (nad6_mafft_trimAl)	K3Pu+F+I+G4

Table S4 Information on each gene fragment of *N. longicornis*.

Name	PX277132					
	**Start**	**Stop**	**Strand**	**Length**	**IGR***	**Codons**
COX1	1	1551	H	1551	2	GTG/TAA
trnS2	1554	1624	L	71	3	
trnD	1628	1699	H	72	7	
COX2	1707	2397	H	691		ATG/T
trnK	2398	2471	H	74	1	
ATP6	2473	2649	H	177	-10	ATG/TAA
ATP8	2640	3322	H	683		ATG/TA
COX3	3323	4107	H	785		ATG/TA
trnG	4108	4177	H	70		
ND3	4178	4526	H	349		ATG/T
trnR	4527	4594	H	68		
ND4l	4595	4891	H	297	-7	ATG/TAA
ND4	4885	6265	H	1381		ATG/T
trnH	6266	6334	H	69		
trnS1	6335	6401	H	67	3	
trnL1	6405	6477	H	73		
ND5	6478	8307	H	1830	-4	ATG/TAA
ND6	8304	8825	L	522		ATG/TAG
trnE	8826	8894	L	69	5	
Cytb	8900	10040	H	1141		ATG/T
trnT	10041	10114	H	74	-1	
trnP	10114	10183	L	70		
NCR	10184	11033	H	850		
trnF	11034	11101	H	68		
12s	11102	12049	H	948		
trnV	12050	12120	H	71		
16s	12121	13818	H	1698		
trnL2	13819	13892	H	74		
ND1	13893	14867	H	975	4	ATG/TAA
trnI	14872	14942	H	71	-1	
trnQ	14942	15012	L	71	-1	
trnM	15012	15081	H	70		
ND2	15082	16127	H	1046		ATG/TA
trnW	16128	16199	H	72		
trnA	16200	16268	L	69	1	
trnN	16270	16342	L	73	36	
trnC	16379	16445	L	67		
trnY	16446	16513	L	68		

* Intergenic Region: Negative numbers indicate overlapping nucleotides between adjacent genes.

Table S5. Base composition of the *N. longicornis* mitochondrial genome.

Regions	PX277132							
	**Size (bp)**	**A%**	**T%**	**C%**	**G%**	**AT(%)**	**AT skew**	**GC skew**
Full genome	16514	29.9	24.9	30.2	15.0	54.8	0.090	-0.335
PCGs	11418	27.0	27.1	31.4	14.5	54.1	-0.003	-0.368
tRNAs	1551	28.9	28.0	20.0	23.1	56.9	0.017	0.072
rRNAs	2646	34.0	19.1	27.1	19.8	53.1	0.281	-0.157
1st codon position	3806	27.3	20.1	28.1	24.5	47.4	0.153	-0.069
2nd codon position	3806	18.3	40.3	27.9	13.5	58.6	-0.376	-0.348
3rd codon position	3806	35.3	21.0	38.2	5.5	56.3	0.255	-0.747
12S rRNA	948	32.9	19.4	27.1	20.6	52.3	0.258	-0.137
16S rRNA	1698	34.6	18.9	27.1	19.3	53.5	0.294	-0.169
ATP6	683	27.5	28.6	31.8	12.2	56.1	-0.018	-0.447
ATP8	177	27.7	23.7	38.4	10.2	51.4	0.077	-0.581
COX1	1551	25.9	28.9	28.5	16.7	54.8	-0.054	-0.261
COX2	691	30.4	25.3	28.7	15.6	55.7	0.091	-0.294
COX3	785	27.6	26.8	30.1	15.5	54.4	0.016	-0.318
Cytb	1141	26.0	28.9	31.2	13.8	54.9	-0.053	-0.385
ND1	975	25.9	25.1	34.3	14.7	51.0	0.016	-0.400
ND2	1046	29.9	23.3	36.0	10.7	53.2	0.124	-0.542
ND3	349	25.5	30.1	31.8	12.6	55.6	-0.082	-0.432
ND4	1381	28.2	24.8	34.4	12.5	53.0	0.064	-0.466
ND4L	297	24.2	25.9	35.0	14.8	50.1	-0.034	-0.405
ND5	1830	29.3	25.6	33.1	12.0	54.9	0.069	-0.469
ND6	522	12.5	42.1	12.1	33.3	54.6	-0.544	0.468
NCR	850	33.4	32.8	19.2	14.6	66.2	0.009	-0.136

Table S6. Codon number and RSCU of *N. longicornis* mitochondrial PCGs.

PX277132							
**AA**	**Codon**	**Count**	**RSCU**	**AA**	**Codon**	**Count**	**RSCU**
Phe	UUU(F)	98	0.85	Tyr	UAU(Y)	35	0.65
Phe	UUC(F)	132	1.15	Tyr	UAC(Y)	72	1.35
Leu2	UUA(L)	64	0.59	SC	UAA(*)	5	3.33
Leu2	UUG(L)	22	0.2	SC	UAG(*)	1	0.67
Leu1	CUU(L)	153	1.41	His	CAU(H)	21	0.38
Leu1	CUC(L)	176	1.62	His	CAC(H)	89	1.62
Leu1	CUA(L)	209	1.92	Gln	CAA(Q)	92	1.86
Leu1	CUG(L)	28	0.26	Gln	CAG(Q)	7	0.14
Ile	AUU(I)	142	1.02	Asn	AAU(N)	36	0.53
Ile	AUC(I)	136	0.98	Asn	AAC(N)	99	1.47
Met	AUA(M)	133	1.53	Lys	AAA(K)	73	1.87
Met	AUG(M)	41	0.47	Lys	AAG(K)	5	0.13
Val	GUU(V)	67	1.35	Asp	GAU(D)	19	0.57
Val	GUC(V)	58	1.17	Asp	GAC(D)	48	1.43
Val	GUA(V)	61	1.23	Glu	GAA(E)	77	1.64
Val	GUG(V)	13	0.26	Glu	GAG(E)	17	0.36
Ser2	UCU(S)	28	0.66	Cys	UGU(C)	11	0.88
Ser2	UCC(S)	91	2.16	Cys	UGC(C)	14	1.12
Ser2	UCA(S)	65	1.54	Trp	UGA(W)	105	1.78
Ser2	UCG(S)	8	0.19	Trp	UGG(W)	13	0.22
Pro	CCU(P)	43	0.77	Arg	CGU(R)	10	0.56
Pro	CCC(P)	101	1.81	Arg	CGC(R)	8	0.44
Pro	CCA(P)	77	1.38	Arg	CGA(R)	48	2.67
Pro	CCG(P)	2	0.04	Arg	CGG(R)	6	0.33
Thr	ACU(T)	48	0.61	Ser1	AGU(S)	9	0.21
Thr	ACC(T)	150	1.91	Ser1	AGC(S)	52	1.23
Thr	ACA(T)	106	1.35	SC	AGA(*)	0	0
Thr	ACG(T)	10	0.13	SC	AGG(*)	0	0
Ala	GCU(A)	54	0.65	Gly	GGU(G)	24	0.4
Ala	GCC(A)	147	1.76	Gly	GGC(G)	81	1.36
Ala	GCA(A)	123	1.47	Gly	GGA(G)	105	1.76
Ala	GCG(A)	10	0.12	Gly	GGG(G)	28	0.47

*Stop codon
