# Supplementary figures and images for: Integrative Taxonomy of Nuchequula longicornis (Teleostei: Leiognathidae) from Chinese Waters: Morphological Analysis, Mitogenomic Characterization, and Phylogenetic Implications
Source: Biology (Basel). 2026 Jan 30;15(3):260. doi: 10.3390/biology15030260 (PMC12897343; doi:10.3390/biology15030260)

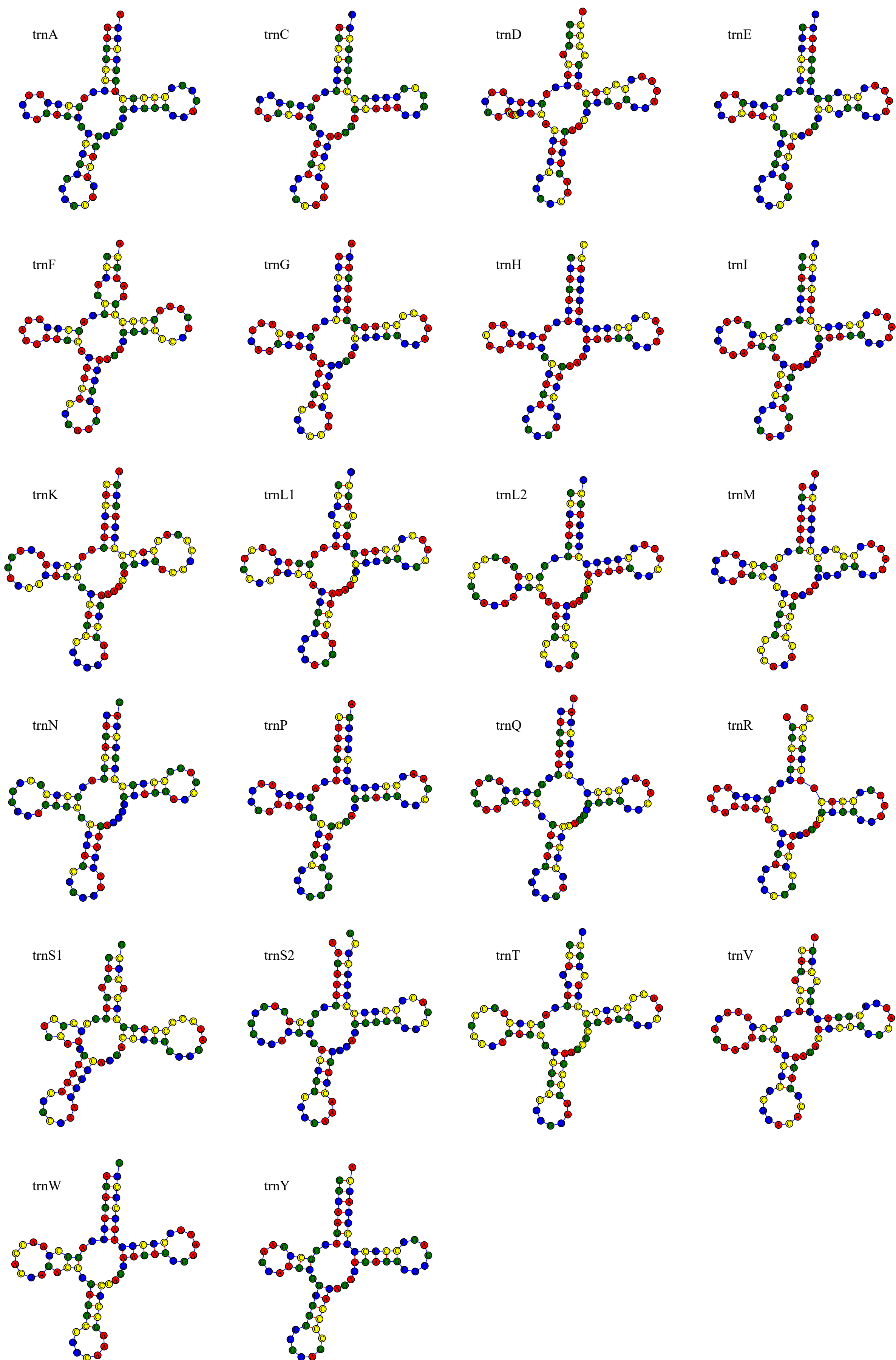

**Figure S2** Secondary structure of the 22 tRNA genes of the mitochondrial genome of *N. longicornis*.

Supplement: Supplementary file 1 [file biology-15-00260-s001.zip › Figure S2 Secondary structure of the 22 tRNA genes of the mitochondrial genome of N. longicornis.pdf]
